# County-Level Factors and Mortality Among Pacific Islander Compared With Asian American Adults

**DOI:** 10.1001/jamanetworkopen.2025.14248

**Published:** 2025-06-06

**Authors:** Jaimie Z. Shing, Paloma R. Mitra, Neal D. Freedman, Kekoa Taparra, Nicole V. DeVille, Jazmyn L. Bess, Jessica M. Madrigal, Amy Berrington de González, Meredith S. Shiels, Jacqueline B. Vo

**Affiliations:** 1 Division of Cancer Epidemiology and Genetics, National Cancer Institute, Rockville, Maryland; 2 Department of Radiation Oncology, Stanford Medicine, Stanford University, Palo Alto, California; 3 Department of Epidemiology and Biostatistics, School of Public Health, University of Nevada, Las Vegas; 4 Division of Genetics and Epidemiology, The Institute of Cancer Research, London, United Kingdom

## Abstract

**Importance:**

Interactions between race and county-level factors associated with mortality, such as employment, education, income, and population density, are understudied among Asian American and Pacific Islander populations.

**Objective:**

To compare all-cause, cancer, and heart disease mortality rates between Pacific Islander and Asian American adults across county-level factors.

**Design, Setting, and Participants:**

This cross-sectional study examined National Center for Health Statistics death certificate data on non-Hispanic Asian American and Pacific Islander adults (aged 20-84 years) between January 1, 2018, and December 31, 2020. County-level socioeconomic data were obtained from the American Community Survey, and population density was determined using Rural-Urban Continuum Codes. Analyses were conducted between August 1, 2023, and September 4, 2024.

**Exposures:**

County-level unemployment, educational attainment, median household income, and population density.

**Main Outcomes and Measures:**

Age-standardized all-cause, cancer, and heart disease mortality rates and mortality rate ratios (MRRs), comparing Pacific Islander with Asian American individuals by sex and age. Interactions between race and county-level factors associated with MRRs were evaluated using *P* value for trend across county-level factors.

**Results:**

During 2018 to 2020, 43 221 696 Asian American and 1 281 221 Pacific Islander adults resided in the US. A total of 148 939 Asian American individuals (16.7% aged 20-54 years, 17.2% aged 55-64 years, and 66.1% aged ≥65 years; 57.5% male) and 9628 Pacific Islander individuals (29.9% aged 20-54 years, 23.0% aged 55-64 years, and 47.1% aged ≥65 years; 57.2% male) died of any cause. Across all county-level factors, Pacific Islander adults had elevated all-cause, cancer, and heart disease mortality rates compared with Asian American adults (female: MRR range from 1.82 [95% CI, 1.67-1.98] for population <250 000 to 2.93 [95% CI, 2.73-3.14] for lowest unemployment tertile; male: MRR range from 1.64 [95% CI, 1.50-1.78] for lowest income tertile to 2.47 [95% CI, 2.31-2.63] for lowest unemployment tertile). Across all county-level factors, the largest relative all-cause mortality differences between Pacific Islander and Asian American adults occurred in counties with the lowest unemployment (female: MRR, 2.93 [95% CI, 2.73-3.14]; male: MRR, 2.47 [95% CI, 2.31-2.63]), highest educational attainment (female: MRR, 2.71 [95% CI, 2.53-2.90]; male: MRR, 2.39 [95% CI, 2.25-2.54]), highest median household income (female: MRR, 2.67 [95% CI, 2.56-2.79]; male: MRR, 2.25 [95% CI, 2.17-2.33]), and highest population density (female: MRR, 2.79 [95% CI, 2.67-2.92]; male: MRR, 2.37 [95% CI, 2.28-2.47]). No trends in relative cancer mortality differences between Pacific Islander and Asian American adults across county-level factors were observed overall except for greater population density among women (<250 000 population: MRR, 1.49 [95% CI, 1.25-1.76; >1 000 000 population, 2.13 [95% CI, 1.95-2.32]). The largest heart disease MRRs for Pacific Islander compared with Asian American individuals occurred among those younger than 65 years, with the greatest relative mortality among those aged 20 to 54 years in counties with the lowest unemployment (female: MRR, 14.21 [95% CI, 9.89-20.04]; male: MRR, 5.75 [95% CI, 4.58-7.15]) and highest educational attainment (female: MRR, 13.69 [95% CI, 9.68-18.94]; male: MRR, 6.17 [95% CI, 5.00-7.54]), median household income (female: MRR, 11.97 [95% CI, 9.55-14.91]; male: MRR, 5.16 [95% CI, 4.49-5.91]), and population density (female: MRR, 11.77 [95% CI, 9.39-14.62]; male: MRR, 5.48 [95% CI, 4.76-6.29]).

**Conclusions and Relevance:**

In this cross-sectional study, all-cause mortality disparities between Asian American and Pacific Islander populations worsened in counties with higher socioeconomic status and greater population density. Historical aggregation of Pacific Islander with Asian American individuals may have misled health improvement efforts, especially for Pacific Islander adults who lived in high socioeconomic and more populated areas.

## Key Points

**Question:**

What is the association of county-level sociodemographic factors with mortality rates for Pacific Islander compared with Asian American adults?

**Findings:**

In this cross-sectional study of 43 221 696 Asian American and 1 281 221 Pacific Islander adults, Pacific Islander adults had elevated all-cause, cancer, and heart disease mortality rates compared with Asian American adults across all county-level factors. The largest relative mortality differences occurred for individuals younger than 65 years, particularly among those living in counties with lower unemployment or higher educational attainment, median income, and population density.

**Meaning:**

These findings suggest that Pacific Islander adults living in higher socioeconomic and more populated areas experience the greatest mortality disparities compared with Asian American adults living in the same areas.

## Teaser

This cross-sectional study examines the association between county-level employment, education, income, and population density and all-cause, cancer, and heart disease mortality rates among Asian American and Pacific Islander adults.

## Introduction

In the US, disparities in mortality exist across area-level factors, including socioeconomic status and urbanicity, and these disparities differ by race and ethnicity.^1,2,3,4^ For example, premature all-cause mortality is elevated with greater county-level unemployment and lower county-level median household income, educational attainment, and population density, but this pattern is most prominent for non-Hispanic Black and non-Hispanic White individuals and less clear for Hispanic individuals.^2^ However, among Hispanic populations, higher all-cause and cause-specific mortality is associated with rural compared with urban residence.^4^ Altogether, these patterns suggest that socioeconomic attributes of one’s geographic residence may contribute to mortality differences by race and ethnicity. Few studies have examined the association between county-level attributes and mortality outcomes among Asian American and Pacific Islander populations.

Asian American and Pacific Islander (including Native Hawaiian) populations are 2 rapidly increasing racial groups in the US,^5^ with large socioeconomic differences between them. For example, Pacific Islander populations have lower median household income, lower educational attainment, and higher unemployment rates than Asian American populations.^6,7^ Studies examining the interaction between race and geographic factors and its association with mortality among disaggregated Asian American and Pacific Islander populations are scarce.^8,9^

In 2018, all states collectively implemented the Office of Management and Budget’s 1997 revised federal race classification standards for the separation of Asian American and Pacific Islander races on death certificates.^8,10^ During 2018 to 2020, the leading causes of death among both Asian American and Pacific Islander populations were cancer and heart disease; however, mortality rates were significantly higher for Pacific Islander compared with Asian American adults, even for COVID-19 mortality, emphasizing the heterogeneity of Asian American compared with Pacific Islander populations.^11,12^ Prior studies examining county-level differences in mortality have historically combined Asian American and Pacific Islander populations,^13,14^ which masks the disparities experienced by Pacific Islander communities. Therefore, understanding the interaction between area-level factors and Asian American and Pacific Islander race is imperative to better identify the racial and socioeconomic differences associated with mortality among these populations. In this study, we compared rates of all-cause, cancer-specific, and heart disease–specific mortality between Asian American and Pacific Islander populations across county-level socioeconomic factors and population density, stratifying by sex and age.

## Methods

### Data Sources

In this ecologic cross-sectional study, we used National Center for Health Statistics death certificate data to identify all deaths among all non-Hispanic Asian American (eg, Asian Indian, Chinese, Filipino) and non-Hispanic Pacific Islander (eg, Native Hawaiian and other Polynesian, Melanesian, Micronesian) individuals aged 20 to 84 years in the US from January 1, 2018, to December 31, 2020 (eFigure 1 in Supplement 1).^10^ This study used deidentified, publicly available death certificate data and did not require institutional review board approval or informed consent in accordance with the Common Rule. The study follows the Strengthening the Reporting of Observational Studies in Epidemiology (STROBE) reporting guideline.^15^

We focused on individuals aged 20 to 84 years to match the age groups provided by the 2000 US Standard Population, which was used to generate age-standardize mortality rates in our study.^16^ Data on race and ethnicity were abstracted from death certificates, which were reported to the funeral director by an informant, usually a relative of the decedent, or by observation.^10^ We included individuals who had ethnicity recorded as non-Hispanic or Latino and nonbridged race recorded as Asian American or Pacific Islander on death certificates.^8^ Population counts by race and ethnicity, sex, and age group were ascertained from 2020 US Census Bureau data.^17^ Asian American population estimates represent 3132 of 3143 (99.6%) total US counties; Pacific Islander population estimates represent 2812 of 3143 (89.4%) total US counties. Thus, our results represent the counties where Asian American and Pacific Islander populations reside.

### Outcomes

The primary outcomes were all-cause mortality, cancer mortality, and heart disease mortality. Causes of death were identified using *International Statistical Classification of Diseases, Tenth Revision* codes C00 to C97 (cancer) and I00 to I09, I11, I13, and I20 to I51 (heart disease). Because our analysis was pooled across the 2018-2020 calendar years, COVID-19 deaths were included in the all-cause mortality outcome.

### County-Level Factors

We examined 4 county-level factors: (1) unemployment (percentage of population aged ≥16 years who were unemployed), (2) educational attainment (percentage of population aged ≥25 years with a bachelor’s degree), (3) median household income (measured in 2021 inflation-adjusted US dollars), and (4) population density (population size of each county).^2,18^ We classified county-level percent unemployed, percent with a bachelor’s degree, and median household income into tertiles based on national population distributions, including all racial groups, across counties using data obtained from the 2017-2021 US Census Bureau’s American Community Survey.^2^ We categorized county-level population density using the following 2013 Rural-Urban Continuum Codes: <250 000 people, 250 000 to 1 000 000 people, and >1 000 000 people.^19^

### Statistical Analysis

For all tertiles of county-level unemployment, educational attainment, and median household income and population density categories, we calculated all-cause, cancer-specific, and heart disease–specific mortality rates per 100 000 person-years and 95% confidence intervals for Asian American and Pacific Islander populations separately, stratified by sex (female and male) and age group (20-54 years, 55-64 years, 65-74 years, and 75-84 years). All mortality rates were age standardized to the 2000 US population.^20^ Mortality rate ratios (MRRs) were calculated for each county-level factor comparing mortality rates of Pacific Islander adults with Asian American adults. Mortality rates and MRRs resulting from death counts of less than 10 were suppressed to prevent reidentification. To identify significant trends in mortality rates, *P* values for trends across tertiles were calculated using Poisson regression for county-level unemployment, education, and median household income and across categories for population density. To identify significant trends in relative mortality differences between Asian American and Pacific Islander populations (ie, MRRs) within county-level unemployment, educational attainment, median household income, and population density, *P* values for trends were calculated by testing the interaction between race and county-level factor.

To examine potential heterogeneity due to multiraciality, we conducted a sensitivity analysis that included all Asian American (n = 44 356 797) and Pacific Islander (n = 1 692 747) individuals regardless of Hispanic or Latino ethnicity. We also conducted a sensitivity analysis stratifying by US region to assess whether results differed in counties with greater population distributions of Asian American and Pacific Islander individuals.

All analyses were conducted using SEER*Stat, version 8.4.2^21^ and R, version 4.3.2 (R Foundation for Statistical Computing). All tests were 2-sided, and *P* < .05 was considered statistically significant. Analyses were conducted from August 1, 2023, to September 4, 2024.

## Results

### All-Cause Mortality by County-Level Factors

**Figure 1.**
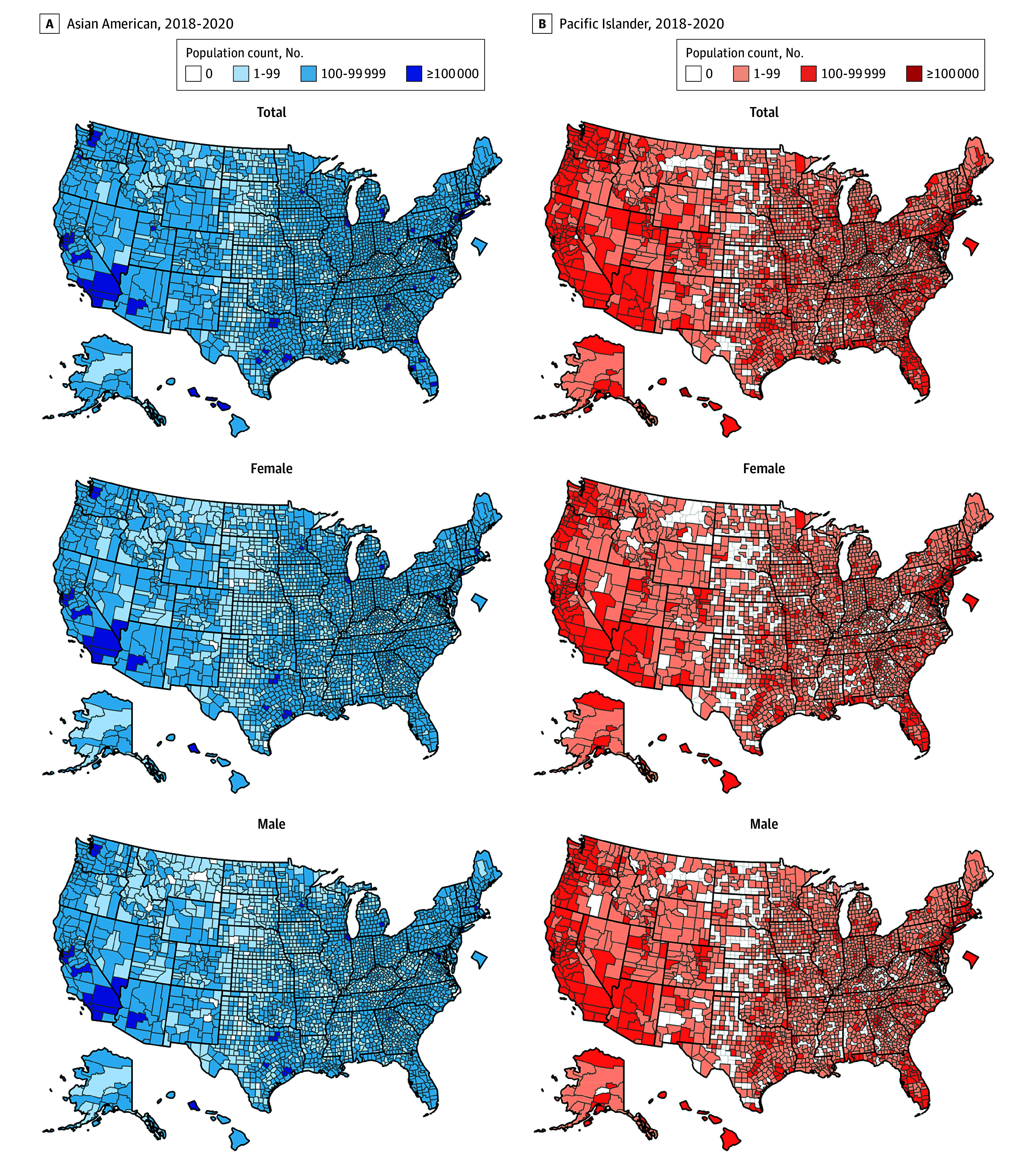
US Population Distribution of Non-Hispanic Asian American and Non-Hispanic Pacific Islander Individuals by US County Thick black lines indicate state boundaries, and thin black lines indicate county geographic boundaries. Asian American population estimates represent 3132 of 3143 (99.6%) total counties; Pacific Islander population estimates represent 2812 of 3143 (89.4%) total counties. Population estimates represent the total person-time contribution during 2018 to 2020, estimated using mid-year population estimates.

During 2018 to 2020, 43 2221 696 Asian American and 1 281 221 Pacific Islander individuals resided in the US, with substantial geographic variation between the 2 groups (eTable 1 in Supplement 2; Figure 1). A large proportion lived in western states (44.2% of Asian American and 74.3% of Pacific Islander individuals), and most were aged 20 to 54 years (70.1 of Asian American and 72.4% of Pacific Islander individuals). A total of 148 939 Asian American individuals (16.7% aged 20-54 years, 17.2% aged 55-64 years, and 66.1% aged ≥65 years; 42.5% female and 57.5% male) and 9628 Pacific Islander individuals (29.9% aged 20-54 years, 23.0% aged 55-64 years, and 47.1% aged ≥65 years; 42.8% female and 57.2% male) died of any cause.

**Figure 2.**
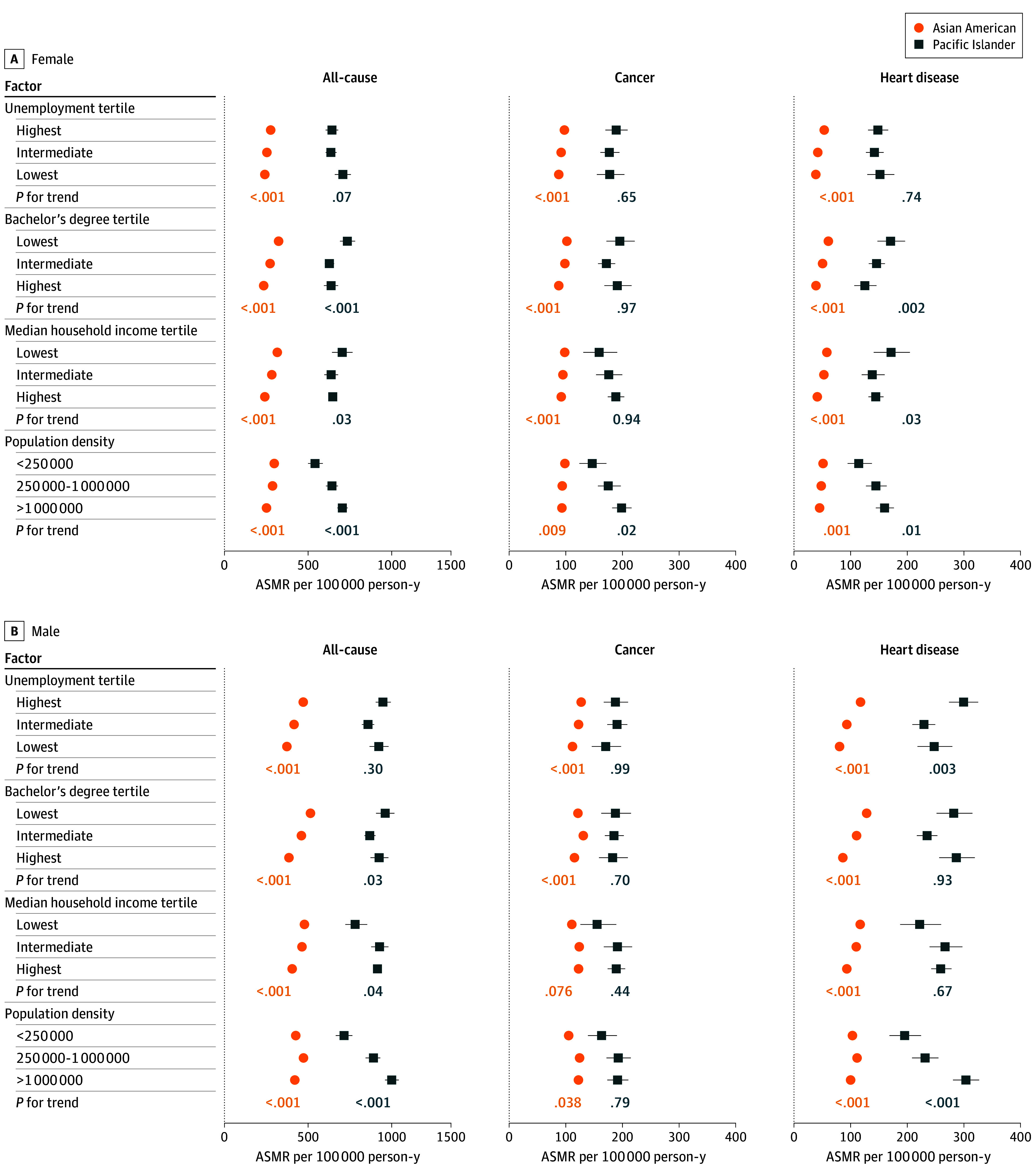
Age-Standardized Mortality Rates (ASMRs) for All-Cause, Cancer-Specific, and Heart Disease–Specific Mortality by Sex Corresponding bounds on point estimates represent 95% confidence intervals. Mortality rates cover 2018 to 2020 and are age-standardized to the 2000 US standard population.

Among female and male Asian American adults, all-cause mortality rates per 100 000 person-years were consistently lower in counties with lower unemployment, higher educational attainment, higher median household income, and higher population density (*P* for trend < .001) (Figure 2). Contrarily, among Pacific Islander men and women, associations between county-level factors and all-cause mortality varied, with lower mortality rates in counties with higher educational attainment (*P* for trend = .03 for men and < .001 for women), higher mortality rates in counties with higher population density (*P* for trend < .001 for both sexes), and no distinct pattern by county-level unemployment. Associations between county-level median household income and all-cause mortality among Pacific Islander individuals differed by sex, with increasing median household income associated with a decrease in all-cause mortality for women (*P* for trend = .03) and an increase among men (*P* for trend .04).

**Figure 3.**
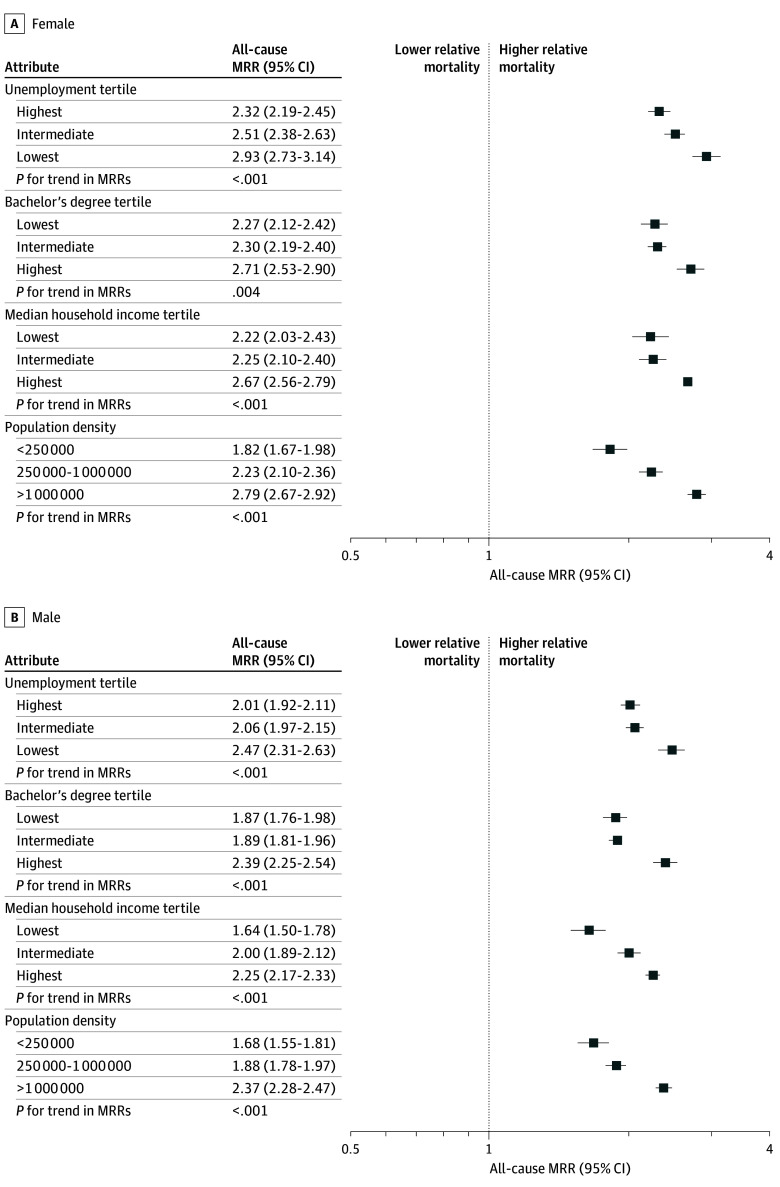
All-Cause Mortality Rate Ratios (MRRs) Between Pacific Islander and Asian American Individuals by Sex The MRRs cover 2018 to 2020 and are age-standardized to the 2000 US standard population.

Across all county-level factors, Pacific Islander adults had elevated all-cause MRRs compared with Asian American adults (female MRR range, from 1.82 [95% CI, 1.67-1.98] for population <250 000 to 2.93 [95% CI, 2.73-3.14] for lowest unemployment tertile; male MRR range, from 1.64 [95% CI, 1.50-1.78] for lowest income tertile to 2.47 [95% CI, 2.31-2.63] for lowest unemployment tertile) (Figure 3). These relative mortality differences (ie, MRRs) were greatest in counties with lower unemployment (*P* for trend < .001 for both sexes), higher educational attainment (*P* for trend = .004 for women and < 0.001 for men), higher median household income (*P* for trend < .001 for both sexes), and higher population density (*P* for trend < .001 for both sexes). The largest relative mortality differences between Pacific Islander and Asian American adults occurred in counties with the lowest unemployment (female: MRR, 2.93 [95% CI, 2.73-3.14]; male: MRR, 2.47 [95% CI, 2.31-2.63]), highest educational attainment (female: MRR, 2.71 [95% CI, 2.53-2.90]; male: MRR, 2.39 [95% CI, 2.25-2.54]), highest median household income (female: MRR, 2.67 [95% CI, 2.56-2.79]; male: MRR, 2.25 [95% CI, 2.17-2.33]), and highest population density (female: MRR, 2.79 [95% CI, 2.67-2.92]; male: MRR, 2.37 [95% CI, 2.28-2.47]) (eTable 2 in Supplement 2). Increased MRR patterns by county-level factors for Pacific Islander populations were consistent when including Hispanic or Latino ethnicity (eTable 3 in Supplement 2). By region, patterns of relative mortality differences across county-level factors were most similar among individuals living in western states (eFigure 2 in Supplement 1). Although there were some similarities among western, southern, and midwestern states, the northeastern states (representing 3.8% of the Pacific Islander population and 20.1% of the Asian American population) lacked power for comparison across many stratifications.

**Figure 4.**
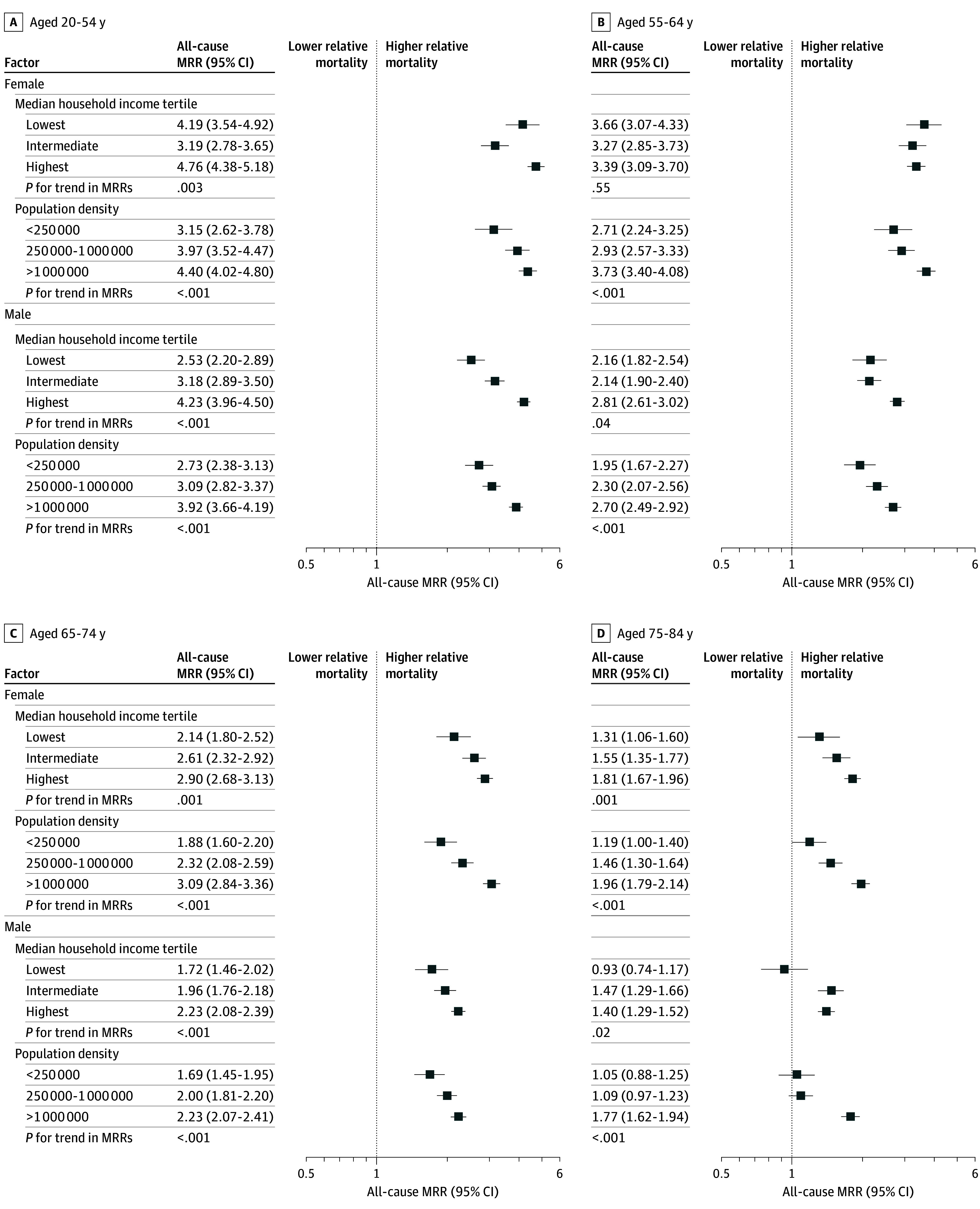
All-Cause Mortality Rate Ratios (MRRs) Between Pacific Islander and Asian American Individuals by Sex and Age Group The MRRs cover 2018 to 2020 and are age-standardized to the 2000 US standard population.

For all county-level factors, relative mortality differences between Asian American and Pacific Islander adults were attenuated among older age groups. The largest relative mortality differences for Pacific Islander compared with Asian American adults were among those aged 20 to 54 years, particularly in counties with the lowest unemployment (female: MRR, 5.17 [95% CI, 4.51-5.89]; male: MRR, 4.44 [95% CI, 4.00-4.91]), highest educational attainment (female: MRR, 4.78 [95% CI, 4.19-5.43]; male: MRR, 4.15 [95% CI, 3.74-4.58]) (eTable 2 in Supplement 2), highest median household income (female: MRR, 4.76 [95% CI, 4.38-5.18]; male: MRR, 4.23 [95% CI, 3.96-4.50]), and highest population density (female: MRR, 4.40 [95% CI, 4.02-4.80]; male: MRR, 3.92 [95% CI, 3.66-4.19]) (Figure 4). Contrarily, the largest absolute mortality differences for Pacific Islander compared with Asian American adults were among those aged 75 to 84 years, most notably in counties with lower unemployment, higher educational attainment, higher median income, and higher population density (female: from 1700.3 to 2081.8 per 100 000; male: from 1285.1 to 2510.1 per 100 000) (eTable 2 in Supplement 2).

### Cancer Mortality by County-Level Factors

Akin to all-cause mortality, Pacific Islander adults experienced persistently higher cancer mortality rates compared with Asian American adults across county-level factors, with notable differences by age and sex (eTable 4 in Supplement 2). However, no trends in relative cancer mortality differences between Pacific Islander and Asian American adults across county-level factors were observed overall, except for a significant association with greater population density among women (<250 000 population: MRR, 1.49 [95% CI, 1.25-1.76; >1 000 000 population, 2.13 [95% CI, 1.95-2.32]; *P* for trend < .001).

### Heart Disease Mortality by County-Level Factors

Across all levels of county-level factors, Pacific Islander adults experienced higher heart disease mortality rates compared with Asian American adults (Figure 2), and MRRs varied by age and sex (eTable 5 in Supplement 2). The largest relative heart disease mortality differences between Pacific Islander and Asian American adults were among those aged 20 to 54 years, particularly in counties with the lowest unemployment (female: MRR, 14.21 [95% CI, 9.89-20.04]; male: MRR, 5.75 [95% CI, 4.58-7.15]), highest educational attainment (female: MRR, 13.69 [95% CI, 9.68-18.94]; male: MRR, 6.17 [95% CI, 5.00-7.54]), highest median household income (female: MRR, 11.97 [95% CI, 9.55-14.91]; male: MRR, 5.16 [95% CI, 4.49-5.91]), and highest population density (female: MRR, 11.77 [95% CI, 9.39-14.62]; male: MRR, 5.48 [95% CI, 4.76-6.29]) (eTable 5 in Supplement 2). Relative heart disease mortality differences were also elevated among individuals aged 55 to 64 years (female MRR range, from 4.29 [95% CI, 2.70-6.64] for <250 000 population to 6.93 [95% CI, 4.75-9.87] for lowest unemployment tertile; male MRR range, from 2.40 [95% CI, 1.74-3.27] for <250 000 population to 5.04 [95% CI, 4.01-6.28] for lowest unemployment tertile), and among men, relative mortality differences were greater in counties with the lowest unemployment (*P* for trend = .006), highest educational attainment (*P* for trend < .001), highest median household income (*P* for trend < .001), and highest population density (*P* for trend < .001).

## Discussion

This cross-sectional study is the first, to our knowledge, to use national death certificate data to examine the interaction between race and county-level factors and its association with mortality among Asian American and Pacific Islander populations. First, associations between county-level factors and mortality differed between Asian American and Pacific Islander populations. Among Asian American adults, lower county-level unemployment, higher educational attainment, higher median household income, and higher population density were associated with lower all-cause, cancer-specific, and heart disease–specific mortality. These findings corroborate prior studies among other racial and ethnic groups, such as aggregated Asian American and Pacific Islander, Hispanic, non-Hispanic Black, and non-Hispanic White populations.^2,4,13,22^ However, by examining Pacific Islander individuals separately in our study, we found that the associations and magnitude of mortality rates varied by county-level factors (eg, no distinct pattern by county-level unemployment, elevated mortality with higher county-level population density). Second, across all factors, Pacific Islander individuals persistently experienced elevated all-cause, cancer-specific, and heart disease–specific mortality rates compared with Asian American individuals. Higher county-level socioeconomic status (lower unemployment, higher educational attainment, higher median household income) and higher population density were associated with significant increases in all-cause MRRs between the populations. Similar patterns with larger magnitudes were also observed for heart disease mortality, but no clear patterns were observed for cancer mortality. Third, relative all-cause, cancer-specific, and heart disease–specific mortality differences between Asian American and Pacific Islander adults were most prominent among those aged 20 to 54 or 55 to 64 years across all county-level factors.

A possible reason for disparate trends may be the differential joint influences of neighborhood-level and individual-level factors by race. Prior studies have reported increased mortality among adults with low individual-level socioeconomic status who lived in high neighborhood-level socioeconomic areas,^23,24,25^ suggesting that community resources available in higher socioeconomic areas (eg, comprehensive health care infrastructures, quality treatment and follow-up care, translational services) may benefit various populations differently.^26^ In general, Pacific Islander adults have lower median household incomes, lower educational attainment, and higher poverty rates compared with Asian American individuals.^7^ This discordance may be further perpetuated for Pacific Islander individuals who may be unable to pay for health care or healthy living activities after accounting for higher costs of living in high socioeconomic status counties. According to the 2020 US Census, most Native Hawaiians now live on the continental US rather than in their ancestral lands of Hawaii due to rising housing costs, possibly resulting from gentrification.^27,28^ The colonization of Hawaii and other US Pacific Island territories (eg, American Samoa, Guam, Northern Mariana Islands) has also contributed to emotional, spiritual, and cultural disconnect; reduced resilience from weakened community ties; and high levels of medical mistrust.^28,29^ These compounding factors may contribute to substantially higher mortality rates faced by Pacific Islander compared with Asian American populations, including the strikingly novel finding of wider disparities in high-income counties.

Another key finding was that the largest relative mortality differences between Pacific Islander and Asian American populations were among individuals younger than 65 years who are ineligible for Medicare, especially for those living in counties with higher socioeconomic status and population density. These disparities may be partly related to limited access to care or no insurance, as the Pacific Islander population has one of the lowest insurance coverage rates in the US.^7^ Even among insured individuals, Pacific Islander adults have significantly poorer health outcomes and greater barriers to care, such as cost,^30^ and may be more likely to delay care compared with Asian American individuals,^31^ potentially contributing to these mortality disparities.

Notably, wide disparities between Asian American and Pacific Islander adults were observed for heart disease mortality, especially for individuals aged 20 to 54 years who lived in higher socioeconomic status and population dense counties, with rates 10 to 13 times higher for Pacific Islander women and 4 to 5 times higher for Pacific Islander men compared with their Asian American counterparts. This finding may be partly due to social and structural stressors among Pacific Islander communities and higher rates of smoking,^32^ hazardous drinking,^33^ and obesity^34^ among young Pacific Islander adults. Additionally, Pacific Islander individuals may live within neighborhoods in high socioeconomic counties that are particularly exposed to food deserts and pollution^35,36,37^ or with low availability of culturally preferred foods and activities.^38,39^

Interestingly, associations between county-level factors and relative cancer mortality differences between Asian American and Pacific Islander populations did not differ overall and for older age groups. A possible reason could be that we included all cancers rather than specific cancer types, as mechanisms for mortality disparities differ by cancer site. A prior analysis found that relative breast cancer survival differences between non-Hispanic Black and non-Hispanic White women worsened with higher census tract socioeconomic status, and the improved breast and prostate survival of the aggregated Asian and Pacific Islander races compared with non-Hispanic White race was attenuated by higher census tract socioeconomic status.^40^ However, for other cancer types (eg, lung, liver, kidney), relative cancer-specific survival differences between racial groups were similar across census tract–level socioeconomic status.^40^

### Strengths and Limitations

Our study’s main strength was the inclusion of all Asian American and Pacific Islander adults in the US from 2018 to 2020, making it the largest study of its kind to date. We were able to estimate robust nationwide mortality rates across county-level factors, revealing striking disparities for Pacific Islander individuals that would have been concealed if aggregated with Asian American individuals. Notably, disaggregation revealed that the age-standardized all-cause mortality rates in 2018 to 2020 among Pacific Islander individuals, which ranged from 695.2 to 1109.7 per 100 000 across county-level attributes, actually surpassed or were comparable to mortality rates of other racial and ethnic groups in 2019, including Hispanic (595.6 per 100 000), non-Hispanic American Indian or Alaska Native (1028.2 per 100 000), non-Hispanic Black (953.5 per 100 000), and non-Hispanic White (802.5 per 100 000) populations.^14^

Our study also had several limitations. First, it included small counts of Pacific Islander individuals for certain strata, lacked self-reported race and ethnicity data, and potentially misclassified cause of death and race on death certificates.^41^ Pacific Islander race could potentially be underreported on death certificates, which would lead to underestimated mortality rates for this population; however, the disparities revealed are potentially unlikely to be due to differences in reporting. Furthermore, there may be differential misclassification of race and ethnicity across county characteristics. For example, areas with higher concentrations of Pacific Islander individuals, such as Hawaiian counties, may have more accurate reporting of Pacific Islander race; however, further research is needed to examine concordance of race by self-report and death certificate reporting in both racial groups. We were limited by an inability to further disaggregate Asian American and Pacific Islander individuals by ethnicity, as both groups represent a heterogeneously diverse population^42,43,44^; such disaggregation may further uncover differences in mortality. Second, the Asian American population has the highest observed income inequality compared with Hispanic, non-Hispanic Black, and non-Hispanic White populations, such that the top 10% of the income distribution earns 10 times as much as the bottom 10%,^45^ and disparities within the Asian American population remain masked through continued aggregation. Use of county-level data also introduces Berkson measurement error,^46^ as socioeconomic factors such as household income were averaged across a county, which may mask the heterogeneity nested within each county. Finally, due to data availability limitations, we were unable to use composite measures,^47,48,49^ such as the Yost Index, to operationalize county-level socioeconomic status, or the Gini Index, which measures income inequality.

## Conclusions

This cross-sectional study found that Pacific Islander individuals faced greater mortality than Asian American individuals across all levels of county-level unemployment, educational attainment, median household income, and population density in 2018 to 2020. These disparities were pronounced among individuals ineligible for Medicare (ie, aged <65 years), particularly in counties with higher socioeconomic status and population density. Historical aggregation with Asian American populations may have misled health improvement efforts away from the hidden disparities faced by Pacific Islander individuals. Consequently, tailored interventions should address the underlying socioeconomic and systemic factors of health care and healthy living associated with poor health outcomes among Pacific Islander communities, both at the individual and neighborhood level, particularly among those living in high socioeconomic areas.

## Notes

Supplement 1. **eFigure 1.** Flow Diagram of Inclusion/Exclusion of Asian American and Pacific Islander Individuals Who Died Between 2018 and 2020 in the US **eFigure 2.** Age-Standardized All-Cause Mortality Rate Ratios per 100 000 Person-Years by Sex and Census Region Among Female and Male Individuals Living in the US

Supplement 2. **eTable 1.** US Population Estimates and Total Deaths for non-Hispanic Asian American and non-Hispanic Pacific Islander Individuals Aged 20-84 Years From 2019-2020 **eTable 2.** All-Cause Mortality Rates, Rate Ratios, and 95% CIs for non-Hispanic Asian American and non-Hispanic Pacific Islander Individuals Overall and by Age Group **eTable 3.** All-Cause Mortality Rates, Rate Ratios, and 95% CIs for Asian Americans and Pacific Islanders by Age Group, Including Individuals of Hispanic/Latino or Unknown Ethnicity **eTable 4.** Cancer Mortality Rates, Rate Ratios, and 95% CIs for non-Hispanic Asian American and non-Hispanic Pacific Islander Individuals Overall and by Age Group **eTable 5.** Heart Disease Mortality Rates, Rate Ratios, and 95% CIs for non-Hispanic Asian American and non-Hispanic Pacific Islander Individuals Overall and by Age Group

Supplement 3.
Data Sharing Statement
