# Supplementary material for: County-Level Factors and Mortality Among Pacific Islander Compared With Asian American Adults
Source: JAMA Netw Open. 2025 Jun 6;8(6):e2514248. doi: 10.1001/jamanetworkopen.2025.14248 (PMC12144625; doi:10.1001/jamanetworkopen.2025.14248)
Supplement: Supplement 1. — eFigure 1. Flow Diagram of Inclusion/Exclusion of Asian American and Pacific Islander Individuals Who Died Between 2018 and 2020 in the US eFigure 2. Age-Standardized All-Cause Mortality Rate Ratios per 100 000 Person-Years by Sex and Census Region Among Female and Male Individuals Living in the US [file jamanetwopen-e2514248-s001.pdf]

Supplemental Online Content

Shing JZ, Mitra PR, Freedman ND, et al. County-level factors and mortality among Pacific Islander compared with Asian American adults. *JAMA Netw Open*. 2025;8(6):e2514259. doi:10.1001/jamanetworkopen.2025.14259

eFigure 1. Flow Diagram of Inclusion/Exclusion of Asian American and Pacific Islander Individuals Who Died Between 2018 and 2020 in the US

eFigure 2. Age-Standardized All-Cause Mortality Rate Ratios per 100 000 Person-Years by Sex and Census Region Among Female and Male Individuals Living in the US

This supplemental material has been provided by the authors to give readers additional information about their work.

© 2025 Shing JZ et al. *JAMA Network Open*.

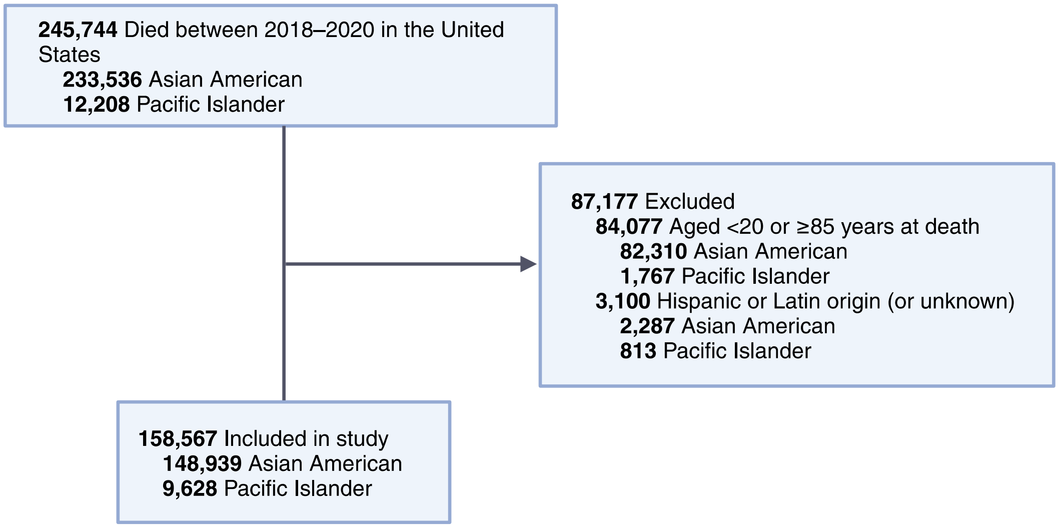

eFigure 1. Flow Diagram of Inclusion/Exclusion of Asian American and Pacific Islander Individuals Who Died Between 2018 and 2020 in the US
Figure created with BioRender.com.

© 2025 Shing JZ et al. *JAMA Network Open*.

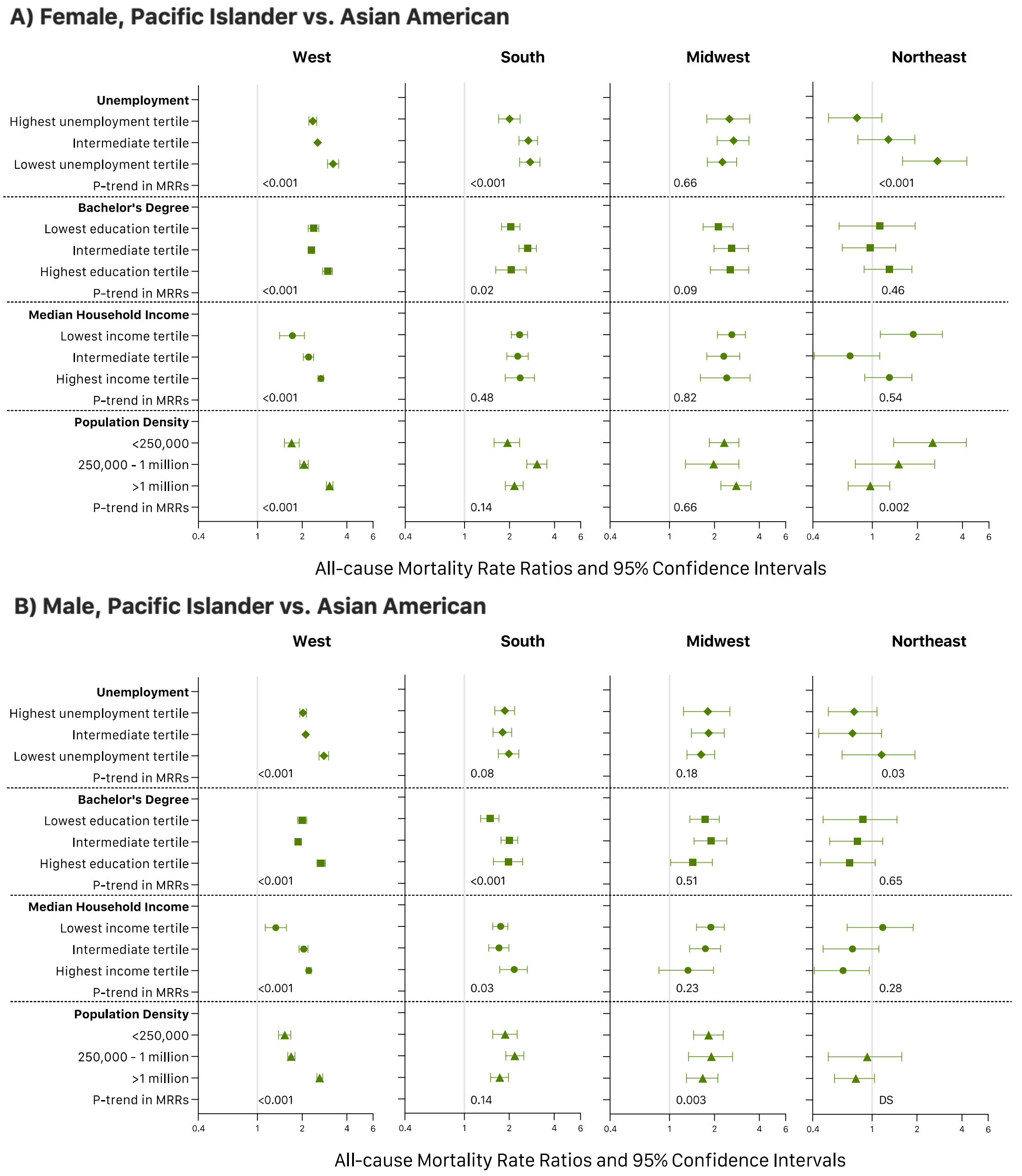

eFigure 2. Age-Standardized All-Cause Mortality Rate Ratios per 100,000 Person-Years by Sex and Census Region[1] Among Female (A) and Male (B) Individuals Living in the US

Asian American and Pacific Islander population lives in 446 counties in the Western region. Midwest represents 1052 of counties. South represents 1421 of counties. Northeast represents 217 of counties.

[1] Census regions consist of the following states:
West (Arizona, Colorado, Idaho, Montana, Nevada, New Mexico, Utah, Wyoming, Alaska, California, Hawaii, Oregon, and Washington)
South (Delaware, District of Columbia, Florida, Georgia, Maryland, North Carolina, South Carolina, Virginia, West Virginia, Alabama, Kentucky, Mississippi, Tennessee, Arkansas, Louisiana, Oklahoma, and Texas)
Midwest (Illinois, Indiana, Michigan, Ohio, Wisconsin, Iowa, Kansas, Minnesota, Missouri, Nebraska, North Dakota, and South Dakota)
Northeast (Connecticut, Maine, Massachusetts, New Hampshire, Rhode Island, and Vermont)

DS = deaths <10 suppressed and p-trend not calculated

© 2025 Shing JZ et al. *JAMA Network Open*.
