# Supplementary material for: County-Level Factors and Mortality Among Pacific Islander Compared With Asian American Adults
Source: JAMA Netw Open. 2025 Jun 6;8(6):e2514248. doi: 10.1001/jamanetworkopen.2025.14248 (PMC12144625; doi:10.1001/jamanetworkopen.2025.14248)
Supplement: Supplement 3. — Data Sharing Statement [file jamanetwopen-e2514248-s003.pdf]

# Data Sharing Statement

Shing. County-Level Factors and Mortality Among Pacific Islander Compared With Asian American Adults. *JAMA Netw Open*. Published June 06, 2025.
doi:10.1001/jamanetworkopen.2025.14248

**Data**
**Data available:** No

**Additional Information**
**Explanation for why data not available:** The data are publicly available from the Centers for Disease Control and Prevention Wide-Ranging Online Data for Epidemiologic Research (CDC WONDER) database: https://wonder.cdc.gov/ucd-icd10-expanded.html.
